# SCIFIO: an extensible framework to support scientific image formats

**DOI:** 10.1186/s12859-016-1383-0

**Published:** 2016-12-07

**Authors:** Mark C. Hiner, Curtis T. Rueden, Kevin W. Eliceiri

**Affiliations:** 1 Laboratory for Optical and Computational Instrumentation, University of Wisconsin at Madison, 271 Animal Science Building, 1675 Observatory Dr., Madison, 53706 WI USA; 2 Morgridge Institute for Research, 330 N. Orchard, Madison, WI USA

**Keywords:** SCIFIO, Image analysis, Open-source, Bio-Formats, ImageJ

## Abstract

**Background:**

No gold standard exists in the world of scientific image acquisition; a proliferation of instruments each with its own proprietary data format has made out-of-the-box sharing of that data nearly impossible. In the field of light microscopy, the Bio-Formats library was designed to translate such proprietary data formats to a common, open-source schema, enabling sharing and reproduction of scientific results. While Bio-Formats has proved successful for microscopy images, the greater scientific community was lacking a domain-independent framework for format translation.

**Results:**

SCIFIO (SCientific Image Format Input and Output) is presented as a freely available, open-source library unifying the mechanisms of reading and writing image data. The core of SCIFIO is its modular definition of formats, the design of which clearly outlines the components of image I/O to encourage extensibility, facilitated by the dynamic discovery of the SciJava plugin framework. SCIFIO is structured to support coexistence of multiple domain-specific open exchange formats, such as Bio-Formats’ OME-TIFF, within a unified environment.

**Conclusions:**

SCIFIO is a freely available software library developed to standardize the process of reading and writing scientific image formats.

## Background

Image formats are defined by the logical layout of metadata and pixel information across one or more data sources. Proprietary file formats (PFFs) are created when an imaging instrument, such as a microscope, records such data in a structure that is not publicly described. PFFs are especially problematic in scientific domains, as each company or even instrument brings the potential for a new file format, possibly requiring licensed software to decode, or the file format changing in structure without notice or recourse. The scientific method necessitates that data can be analyzed by others to verify and reproduce results; when said data is stored in a proprietary format, by definition, it cannot be freely shared and inspected.

In response to the proliferation of PFFs in the fields of life science, the Open Microscopy Environment (OME) consortium developed the Bio-Formats library to standardize the reading of microscopy data [1]. Bio-Formats provides an application program interface (API) for reading and writing images, backed by a comprehensive collection of extensions to decode format-specific information and translate it into an open specification called the OME data model [2]. A translated image can then be written as OME-TIFF, an “open-exchange format” which combines the universal readability of the TIFF standard with an XML schema representing the OME data model (OME-XML). These OME-TIFF images can be freely shared, with pixel data accessible via standard libraries such as libtiff [3], and the complete metadata parseable by any standards-compliant XML reader. In this way, the Bio-Formats project greatly mitigates the PFF problem in microscopy.

Bio-Formats has become an essential tool for scientists worldwide; however, its metadata model specifically targets 5-dimensional images in microscopy and related life sciences disciplines. PFFs from other scientific domains—e.g., medical imaging, astronomy, industrial x-rays, materials science and geoscience—each have their own unique considerations with respect to the dimensionality and metadata of their images; as such, it would be infeasible for a single “one-size-fits-all” metadata model to fully address the needs of scientific imaging as a whole. With this conclusion in mind, we have developed the SCIFIO (SCientific Image Format Input and Output) library, generalizing the success of Bio-Formats to create a domain-independent image I/O framework enabling seamless and extensible translation between image metadata models. The goal of SCIFIO is to provide the architecture that will equally facilitate: 1) the conversion of additional formats into supported open-exchange formats such as OME-TIFF and 2) the integration of additional scientific open-exchange formats such as Digital Imaging and Communications in Medicine (DICOM) [4], Flexible Image Transport System (FITS) [5] and netCDF [6] into a common image I/O framework.

## Implementation

SCIFIO is implemented as a plugin suite for the SciJava plugin framework. Its core is written under the permissive BSD license to maximize freedom of inclusion in both open and closed source applications. The SciJava framework collects *Plugins* in an application *Context* which are typically accessed via *Services*. As such, SCIFIO defines a collection of *Plugins* and *Services* facilitating image I/O. Developers will typically start with the *SCIFIO* class itself: a *Gateway* to the SciJava *Context* providing convenient accessor methods for functional components of the SCIFIO framework.

The SciJava framework sorts *Plugins* by “type,” representing the role of a given *Plugin*. Extensibility and flexibility is achieved by providing a public *Service* API which organizes and delegates to available *Plugins* of each type. Thus, SCIFIO development is primarily concerned with adding new *Plugin* implementations to achieve a desired result. The following sections describe the key *Plugin* types in SCIFIO, and the behavior they control.

**Fig. 1:**
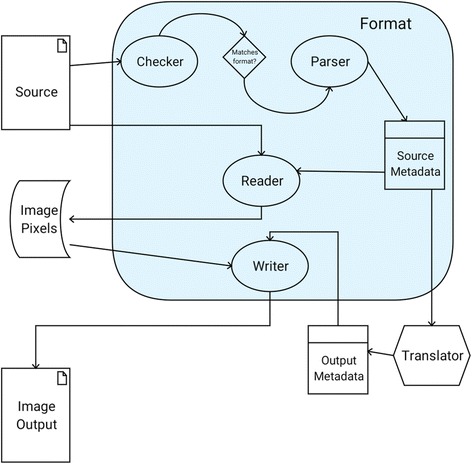
Components of a *Format* plugin and their role in image I/O

First and foremost is the *Format. Formats* are a collection of interface-driven components (Fig. 1) defining the steps for decoding an image source to its metadata and pixel values. In SCIFIO, the ImageJ Common data model is used to describe pixels; this data model is built on ImgLib2 [7] due to its type and algorithmic flexibility, ensuring images opened with SCIFIO are universally recognized within the ImageJ ecosystem [8]. A *Format* must always include a *Metadata* component defining its unique fields and structures, such as acquisition instrument details, dimensional axis types, or detector emission wavelengths. Each *Metadata* implementation must also be able to express itself as a standard format-independent *ImageMetadata* object, establishing a common baseline for use within the framework.

The *Checker* component contains the logic for matching a given *Format* with a potential image source, while the *Parser* component performs the actual creation of *Metadata* from that source. The *Reader* and *Writer* components use *Metadata* to read or write pixel data, respectively. Given the goal of freely shareable image data, *Writers* are optional components and should not be implemented for proprietary formats.

**Fig. 2:**
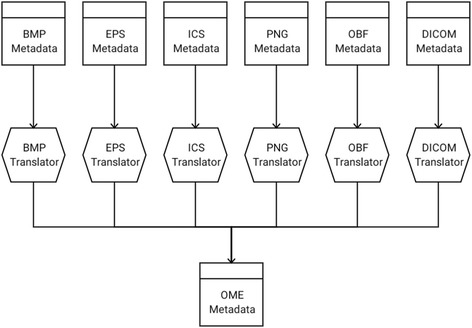
SCIFIO-OME-XML *Translator* suite, for converting metadata to the OME-TIFF open-exchange format

A second essential *Plugin* type is the *Translator*, which encodes logic for conversion from one *Metadata* type to another. *Translators* enable the standardization of proprietary formats to common *Metadata* structures such as OME, and hence play a key role in converting images between *Formats. Translators* are typically created to accompany *Writers*, ensuring *Format*-specific metadata is properly populated. Additionally, the *Translator* framework enables the integration of new open-exchange formats via *Translator*-only libraries, converting supported *Metadata* types to the new standard. An example of this model can be seen in the SCIFIO-OME-XML component (Fig. 2).

**Fig. 3:**
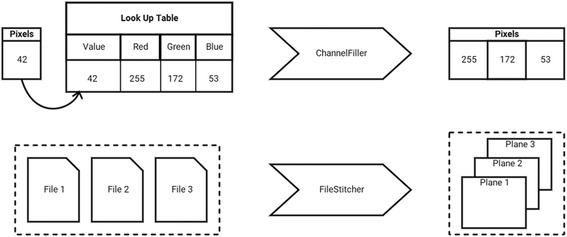
Behavior of *ChannelFiller* and *FileStitcher Filter* plugins

While *Formats* and *Translators* add new behavior to the base framework, SCIFIO also has *Plugin* types to control existing behavior. For example, *Filter* plugins provide a *Format*-agnostic mechanism for modifying *Reader* behavior. *Filters* create an ordered chain of delegation, each operating on the data of its parent, and can be individually toggled ‘on’ or ‘off’ on a per-*Reader* basis. Sample *Filter* stacking behavior is illustrated in a *ChannelFiller* for converting “indexed color” pixels to RGB values and a *FileStitcher* for unifying multiple files on disk to form one dataset (Fig. 3).

With all SciJava *Plugins*, a numeric priority value attached to each class creates an implicit relative ordering for operations—e.g., order of *Checker* querying, *Translator* querying, or *Filter* application. Priorities are automatically considered when using the SCIFIO *Services*: from the *FormatService* polling *Checker* components to the *TranslatorService* finding the correct *Translator* for a given request, priorities allow querying the most specific solutions first, before moving to more general options. These pieces together provide a robust and flexible library for reading and writing image data.

## Results and discussion

As the fundamental goal of SCIFIO is to establish an extensible framework for image support, the SciJava framework is a logical choice for implementation. SciJava provides extensible solutions to common software problems, which implicitly benefit SCIFIO. A core example is the extensible script language framework (http://imagej.net/Scripting) which effectively allows SCIFIO to be used from any number of programming languages without requiring language-specific considerations in SCIFIO itself.

ImageJ [9] presents the flagship use case for SCIFIO, allowing an established community to vet and refine the library. Although users do not directly interact with SCIFIO API, all image I/O operations in ImageJ ultimately rely on SCIFIO. As developers contribute new *Format* plugins for image types relevant to their work, any application using SCIFIO can immediately benefit from the new plugin. Looking beyond ImageJ, projects like KNIME Image Processing (KNIP), built on the KNIME Analytics Platform [10], have already adopted SCIFIO for their image I/O mechanism. This sort of code sharing leads to a form of mutualistic collaboration: a new *Format* plugin developed for KNIP will automatically work in ImageJ, with the converse true as well. Equally importantly, both ImageJ and KNIP can implicitly operate on image data produced by the other program, laying the foundation for algorithmic interoperability.

Collaborations like this would not be possible with a focused library like Bio-Formats. KNIME is a platform for extensible workflows, thus its handling of image data demands flexibility beyond the fixed 5D microscopy schema of OME. Additionally, Bio-Formats’ mechanism of format extension requires either modification of a text-based configuration file to define format priority, which can lead to conflicts if multiple libraries provide differing versions of this file, or runtime modification by API calls, which may not be reproducible without a central mechanism controlling these calls. Conversely, the dynamic discovery of the SciJava plugin framework allows SCIFIO developers to provide their *Formats* completely independently—e.g., on an ImageJ, KNIME or Eclipse update site, while SCIFIO’s backing by the ImageJ Common data model ensures adaptation to any future requirements in imaging dimensionality and data types.

Bio-Formats readers and writers and SCIFIO *Format* components define similar high level logic, but in Bio-Formats several I/O steps are conflated in a single monolithic interface with many protected methods as potential extension points. SCIFIO encapsulates each I/O step into its own dedicated component, to minimize the effort required in format development. Whether a format is added to Bio-Formats or SCIFIO libraries; the SCIFIO-BF-Compat and SCIFIO-OME-XML components offer bidirectional compatibility between SCIFIO and Bio-Formats.

Bio-Formats has demonstrated the feasibility of standardizing a broad field of PFFs into a common open-exchange format. SCIFIO provides a natural generalization of thinking, allowing extension to new domains, through the integration of their *Metadata* standards and open-exchange formats via *Translators*, and clear paths for contributing to existing domains by encapsulating the logic of *Format* components. Given the added immediate power of the Bio-Formats integration layers, we see the SCIFIO framework as a potential unifying solution to PFFs in scientific image data.

## Conclusions

SCIFIO is an open-source library generalizing the successful structure of Bio-Formats to create a domain-independent framework for the reading, writing, and translation of images. The extensible design of SCIFIO facilitates community contribution, the establishment of domain-specific metadata standards, and integration into a unified system capable of adapting to the demands of scientific imaging analysis.

## Abbreviations

API: Application program interface
I/O: Input and/or output
KNIME: Konstanz Information Miner
KNIP: KNIME Image Processing
OME: Open Microscopy Environment
PFF: Proprietary file formats
SCIFIO: SCientific Image Format Input and Output
